# Modeling the Offensive-Defensive Interaction and Resulting Outcomes in Basketball

**DOI:** 10.1371/journal.pone.0144435

**Published:** 2015-12-14

**Authors:** Leonardo Lamas, Felipe Santana, Matthew Heiner, Carlos Ugrinowitsch, Gilbert Fellingham

**Affiliations:** 1 Faculty of Physical Education, University of Brasilia, Brasilia, Distrito Federal, Brazil; 2 School of Physical Education and Sport, University of Sao Paulo, Sao Paulo, Sao Paulo, Brazil; 3 Department of Applied Mathematics and Statistics, University of California, Santa Cruz, Santa Cruz, California, United States of America; 4 Department of Statistics, Brigham Young University, Provo, Utah, United States of America; Research Center for Sports Sciences, Health and Human Development (CIDESD), University of Trás-os-Montes e Alto Douro, Vila Real, Portugal, PORTUGAL

## Abstract

**Purpose:**

We analyzed the interaction between offensive (i.e. space creation dynamics -SCDs) and defensive (i.e. space protection dynamics—SPDs) actions in six play outcomes (free shot, contested shot, new SCD, reset, foul, and turnover) in Spanish professional basketball games.

**Method:**

Data consisted of 1548 SCD-SPD-outcome triples obtained from six play-off games. We used Bayesian methods to compute marginal probabilities of six outcomes following five different SCDs. We also computed probabilities of the six outcomes following the 16 most frequent SCD-SPD combinations.

**Results:**

The pick action (e.g. pick and roll, pop and pop) was the most prevalent SCD (33%). However, this SCD did not produce the highest probability of a free shot (0.235). The highest probability of a free shot followed the SCD without ball (0.409). The pick was performed not only to attempt scoring but also to initiate offenses, as it produced the highest probability leading to a new SCD (0.403). Additionally, the SPD performed influenced the outcome of the SCD. This reinforces the notion that the opposition (offensive-defensive interaction) should be considered. To the best of our knowledge, in team sports, this is the first study to successfully model the tactical features involved in offense-defense interactions. Our analyses revealed that the high frequency of occurrence of some SCDs may be justified not only by an associated high probability of free shots but also by the possibility of progressively create more space in the defense (i.e. a new SCD as outcome). In the second case, it evidences offensive strategic features of progressive disruption of the defensive system through the concatenation of subsequent offensive actions.

## Introduction

Basketball strategies are designed based on the coaching staff’s expected probability of success of the specified set of plays. Match analysis techniques provide quantitative evidence of the adequacy of the strategy, which is often based on box-score information. The information derived from box-scores (e.g. effective field goal percentage; turnover percentage; offensive and defensive rebound percentage; free throw efficiency) has been systematically improved over the years as researchers have sought to quantify the relationship between these indices and the probabilities of winning matches [1–5]. Nonetheless, these box-score derived indices are based on the frequency of outcomes that occur at the end stages of ball possessions, limiting coaches’ ability to fully understand the causes of the quantified outcomes [6, 7]. Thus, the actual playing pattern of a team may not be fully understood, increasing the error rate in the coach’s decision process [8].

Alternatively, we have seen a trend toward the development of analytical tools that are sensitive to the team’s dynamics (e.g. the patterns of ball circulation and players actions) and the outcome obtained, with the aim of predicting a team’s performance [9–11]. Besides these analytical improvements, the relationship between a priori planning and execution of players actions has been modeled [12]. According to this model, a priori planning, henceforth called team strategy, was defined as a discrete dynamic system, which is described by a set of states. Each state of this dynamic system is described by categorical variables containing tactical specifications that are sufficient to characterize different game circumstances (e.g. actions directly related to the ball).

The annotated states are restricted to a time interval in which the observed tactics (i.e., individual or collective team players actions) have a high influence on team success. In basketball, this interval occurs when the offensive team tries to create space in the opponent’s defensive system to obtain a shooting opportunity. Usually, basketball teams have a limited number of offensive sets, characterized by specific sequences of states, that they use multiple times in a match [13]. In these sequences of states, there exists a large but finite set of possible actions (e.g. offensively: pick and roll; defensively: trap on ball player) that can be performed to achieve a limited set of outcomes (e.g. a free shot, a turnover). Thus, the relationship between sequences of states a team performs and the final outcome, on each play, is affected by the opponent’s actions. Consequently, one should aim to assess the content of the offense-defense interaction to interpret the possible causes of the achieved outcome.

Categorization of offensive and defensive tactical classes of actions in basketball was developed, respectively, by Lamas et al. (2011) and Santana et al. (2015). These authors defined sets of equivalence classes of actions that can be planned and, consequently, performed to create and protect space in basketball matches, denominated as space creation dynamics (SCDs) and space protection dynamics (SPDs), respectively. The compromise between increasing tactical resolution through labeling players’ actions and limiting sample size as a consequence of manual annotation dependency was positively solved as classes demonstrated to satisfatorily discriminate between analyzed teams [13–15].

An extension of previous work on SCD and SPD classes is the application of statistical models that take into consideration the sequential nature of basketball match events to determine the probability distribution of actions leading to positive or negative outcomes, given the actions performed by the adversary. Thus, considering the SCD-SPD-outcome triple can be an interesting approach to represent the basic structure of offensive-defensive interactions in the match and to understand match dynamics. Therefore, the present work aims to analyze the performance in professional basketball matches through modeling the interaction between the SCDs, SPDs and outcomes obtained.

## Materials and Methods

### Sample

The data were gathered using video from six consecutive matches from the same team (Barcelona F.C.), in the same tournament (Liga ACB—Spanish championship, 2010–2011), against semi-final and final adversaries (i.e. Caja Laboral and Bilbao Biskaia). SCD-SPD couples were noted every time they occurred, along with the outcome. Ball possessions that terminated before any interaction occurred were not considered. The Ethical Committee of the School of Physical Education and Sport of the University of Sao Paulo approved all experimental procedures (protocol: 2009-10). S1 Dataset.

### SCD and SPD classes

**Fig 1:**
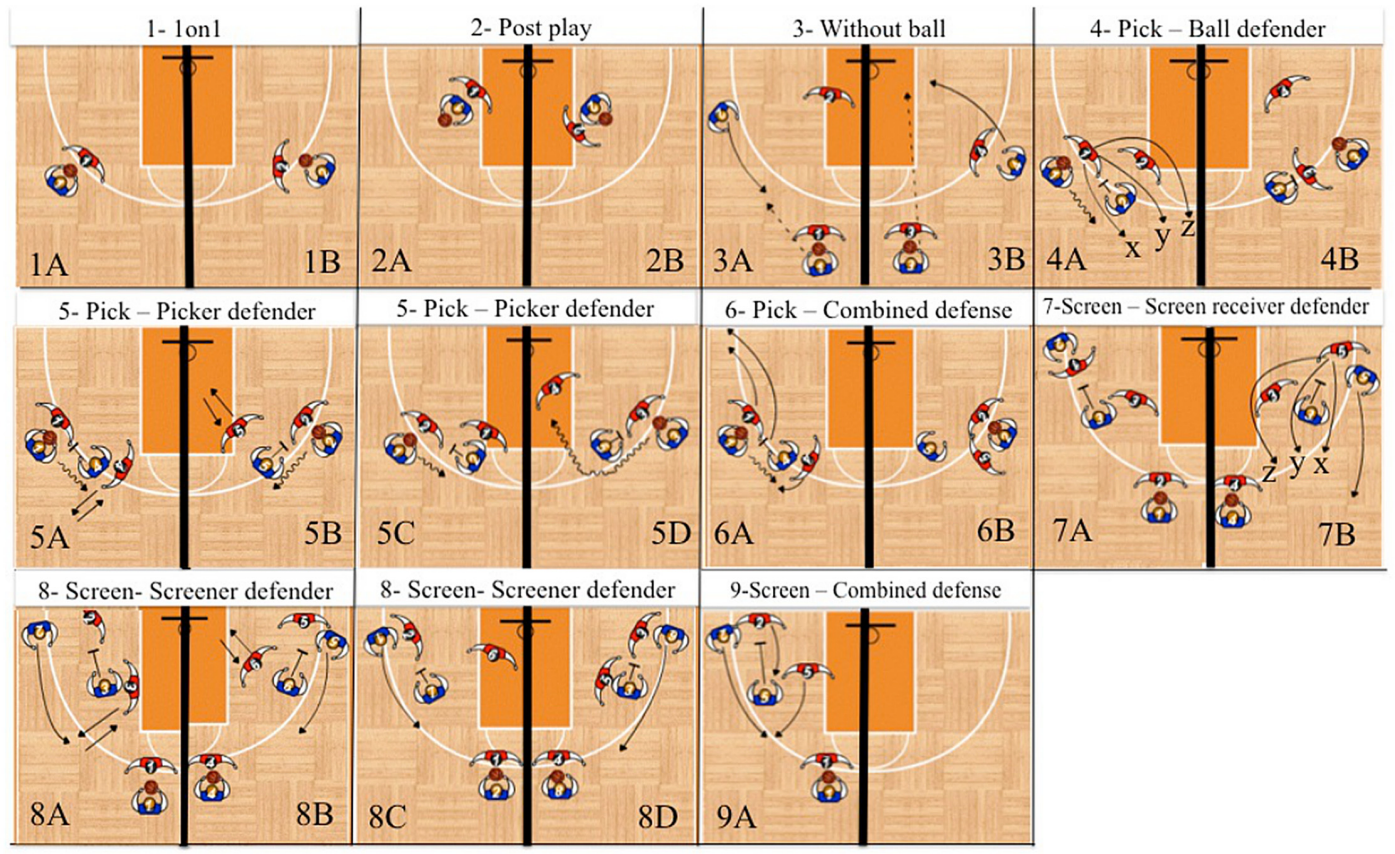
Patterns of SCD-SPD couples in 1 on 1, 2 on 2, and 3 on 3 interactions. Continuous arrows indicate player movement without the ball, zigzag arrows indicate player movement with the ball, dotted arrows indicate a pass, and a T indicates a screen. Diagrams 1A and 1B show a 1 on 1 SCD on the perimeter where the SPD position is neutral in 1A and oriented in 1B. Diagrams 2A and 2B show a 1 on 1 SCD in the post where the SPD position in 2A is neutral and in 2B is oriented. Diagrams 3A and 3B show 2 on 2 interactions where the SPD emphasis is on the player without the ball. In 3A the SPD position is away and in 3B the SPD position is close. Diagrams 4A and 4B show the pick SCD with SPD positions focusing on the ball handler. In 4A—“x” the SPD is second (fight through), in 4A—“y” the SPD is third (middle), and in 4A—“z” the SPD is fourth (behind). In diagram 4B the SPD position is deny. In diagrams 5A-D the SPD defender actions focus on the player setting the screen. In 5A the SPD position is show, in 5B the SPD position is open, in 5C the SPD position is sustain, in 5D the SPD position is away. Diagrams 6A and 6B present combined defense between defenders of ball handler and screener. In 6A and 6B, the SPD positions are, respectively, switch and trap. In diagrams 7–9 the SCD is a screen. In 7A-B the SPD positions focus on the player for whom the screen is being set. In 7A the SPD position is deny. In 7B—“x” the SPD position is second (fight through), in 7B—“y” the SPD position is third (middle), in 7B—“z” the SPD position is fourth (behind). In 8A-D the SPD positions focus on the player setting the screen. In 8A the SPD position is show, in 8B the SPD position is open, in 8C the SPD position is away, in 8D the SPD position is sustain. Diagram 9A displays combined defense between defenders of the player on whom the screen is being set and the screener. The SPD position is the switch. Adapted from Santana et al. (2015), for illustrative purposes only.

Lamas et al. (2011) presented a set of offensive classes, which describes the possible actions used to create space in the adversary’s defensive system. These offensive classes or SCDs were used as a reference to propose corresponding defensive classes or SPDs [14]. In these studies, there were defined equivalent classes that encompass all possible variations of the action that define a given SCD or SPD class. For this purpose, authors followed a very rigorous validation process. Herein, the sampling structure considered an offensive action (i.e. an SCD) as a trigger event for a sequence of interest (i.e. offense-defense-outcome). Thus, the execution of a SPD was conditioned by the occurrence of a previous SCD. Fig 1 provides a pictorial view of the SCD-SPD couples.

In some cases the SPD will be described by two words. For example, the defense of a pick could be described as a second (the defensive action on the ball handler) show (the defensive action on the screener). Another defensive action that we characterize but do not show in Fig 1 is defense out of position.

### Outcomes

All possible outcomes of an SCD-SPD couple were grouped in six classes, a) free shot—the shooting player is not closely guarded, b) contested shot—the shooting player is guarded, c) new SCD—the previous SCD is concatenated with a subsequent SCD, d) reset—offense is neutralized and needs to re-start with a new play, e) foul—the offensive player is fouled, and f) turnover—the offense loses ball possession.

### Reliability procedures

In the experiment, a single researcher collected all sample data. His reliability in assigning an SCD, an SPD and an outcome in a given offense-defense interaction was assessed. To evaluate the intra-observer consistency a set of 200 offensive-defensive couples of actions were randomly selected from ball possessions of high-level basketball games from different leagues (e.g. NBA, EuroLeague). The game analyst annotated each of the triple elements (i.e. offensive action, defensive action and outcome) and classified them according to the SCD, SPD and outcome classes, on three different occasions, one week apart each other. Reliability ratios were evaluated according to the levels of agreement for the Kappa value [16]: <0 less than the chance agreement, 0.01—0.20 slight agreement, 0.21—0.40 fair agreement, 0.41—0.60 moderate agreement, 0.61—0.80 substantial agreement and 0.81—0.99 almost perfect agreement.

### Data Analysis

Analysis was conducted using 1,548 sequences, each consisting of an SCD-SPD-outcome triple. All sequences were pooled from the six matches to obtain adequate counts for statistical inference and were analyzed from two perspectives using Bayesian methods. The first perspective considered the marginal association of SCDs to outcomes, while the second perspective related SCD-SPD combinations to outcomes.

In the first analysis (perspective), counts of SCDs leading to each of the six outcomes were tabulated. For example, the free shot outcome had 77 sequences that were initiated with the 1 on 1 perimeter SCD, 116 sequences initiated with the on ball screen SCD, and so on for each of the five distinct SCDs. These counts of different SCDs leading to a free shot were then modeled as a multinomial random vector in which *n*_1_, *n*_2_, …, *n*_5_ represent these counts, and *p*_1_, *p*_2_, …, *p*_5_ represent the probabilities of each SCD preceding a free shot. Specifically, *p*_1_ is the probability that a 1 on 1 perimeter SCD leads to a free shot with corresponding *n*_1_ = 77, *p*_2_ is the probability that a 1 on 1 post leads to a free shot with corresponding *n*_2_ = 22, etc. The multinomial model is then given by Eq 1:

$$f\;\left(n|p\right)=\frac{(\Sigma_{i=1}^{5}n_{i})!}{\Pi_{i=1}^{5}n_{i}!}p_{1}^{n_{1}}p_{2}^{n_{2}}p_{3}^{n_{3}}p_{4}^{n_{4}}p_{5}^{n_{5}}$$ (1)

where 0 ≤ *p*_*i*_ ≤ 1 for all *i* and $\sum_{i=1}^{5}p_{i}=1$

Posterior inference for the *p*_*i*_ requires a prior distribution over these parameters. A Dirichlet prior was chosen for this model. The density for this prior is given by Eq 2:

$$\pi \left(p|\alpha \right)=\frac{\Gamma (\Sigma_{i=1}^{5}\alpha_{i})}{\Pi_{i=1}^{5}\Gamma \left(\alpha_{i}\right)}p_{1}^{\alpha_{1}-1}p_{2}^{\alpha_{2}-1}p_{3}^{\alpha_{3}-1}p_{4}^{\alpha_{4}-1}p_{5}^{\alpha_{5}-1}$$ (2)

where *α_i_* > 0 and Γ() is the gamma function. The Dirichlet prior is computationally convenient in a multinomial model because of conjugacy, that is, application of Bayes’ theorem over (1) [17] and (2) yields a posterior density of the form presented in Eq 3:

$$\pi \left(p|n,\alpha \right)=\frac{\Gamma (\Sigma_{i=1}^{5}\left(n_{i}+\alpha_{i}\right))}{\Pi_{i=1}^{5}\Gamma \left(n_{i}+\alpha_{i}\right)}p_{1}^{n_{1}+\alpha_{1}-1}p_{2}^{n_{2}+\alpha_{2}-1}p_{3}^{n_{3}+\alpha_{3}-1}p_{4}^{n_{4}+\alpha_{4}-1}p_{5}^{n_{5}+\alpha_{5}-1}$$ (3)

which is again Dirichlet with updated *α* parameters. In this model, the *α* parameters can be considered as prior “counts,” where larger values of *α*_*i*_ weight the prior density in favor of *p*_*i*_. Small values of *α* correspond to a weak prior, resulting in the counts in the data, the *n*_*i*_, dominating the posterior inference for the *p*_*i*_.

In this analysis, the values *α*_*i*_ = 0.098 for *i* = 1, …, 5 were chosen in accordance with recommendations by de Campos and Benavoli (2011), who presented a method of finding weak prior *α* values for which Bayes point estimators of the *p*_*i*_ are comparable to the maximum likelihood estimator in mean squared error. Furthermore, the five *α*_*i*_ values were set equal to one another so as to not favor any one *p*_*i*_ over the others *a priori*.

**Fig 2:**
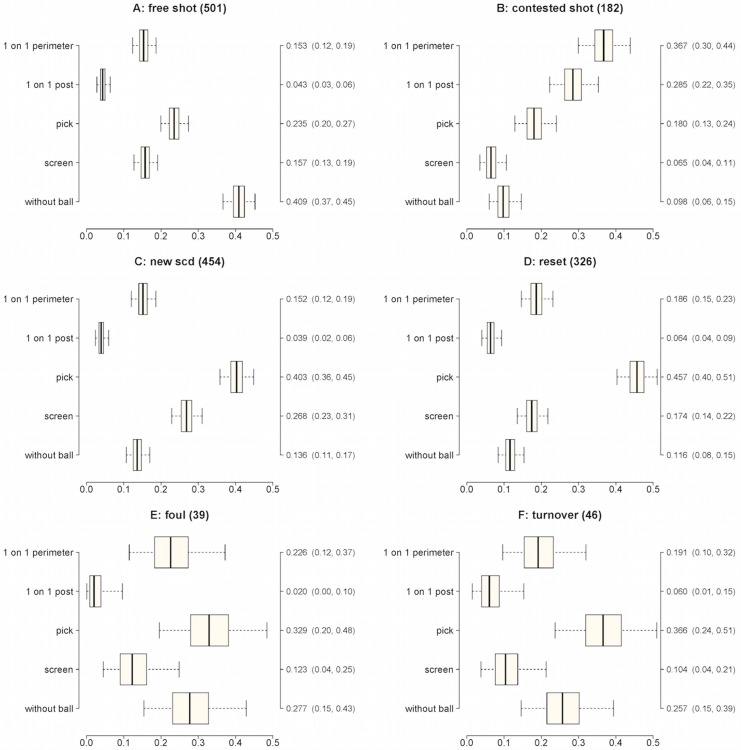
Plots showing point and interval estimates of the probability of each SCD leading to a certain outcome. Plots show median, 50 and 95% credible intervals (CI). Medians and 95% credible intervals are shown on the right axis.

This model was replicated six times, with one model for each outcome (i.e. free shot, contested shot, etc.). The estimated parameters in each model represented the probabilities of each of the five SCDs preceding the specified outcome. In each model, comparative inference for the *p*_*i*_ parameters was carried out using Monte Carlo simulation from the full joint posterior (Eq 3). Posterior simulations used 50,000 independent draws, resulting in a standard deviation of less than 0.003 in Monte Carlo approximations of posterior probabilities. Point and interval estimates for the individual *p*_*i*_ parameters were obtained from their exact marginal posteriors, which are beta distributions [18, 19]. These marginal posterior distributions for all six models are summarized with box plots in Fig 2. Each box plot shows the posterior median point estimates and 95% credible intervals for the *p*_*i*_ are reported next to the box plots. Note that the numbers reported at the top of each plot near the title indicate the total number of data points contributing to the corresponding model.

In the second analysis, counts of outcomes resulting from each SCD-SPD combination were tabulated. For example, the SCD-SPD combination 1 on 1 post (SCD) to out of position (SPD) was followed by a free shot in *n*_1_ = 7 sequences, a contested shot in *n*_2_ = 2 sequences, and so on for the six outcomes. These six counts were modeled as a multinomial vector with parameters *p*_1_, …, *p*_6_, which are interpreted as the probabilities of the 1 on 1 post with an out of position defense combination leading to each of the six outcomes. The same method was employed in this analysis as in the first, with prior *α* = 0.061 adjusted to reflect use of six parameters. This model was replicated for each SCD-SPD combination. Only the couples with frequencies of occurrence ≥ 25 events were used for statistical modeling.

**Fig 3:**
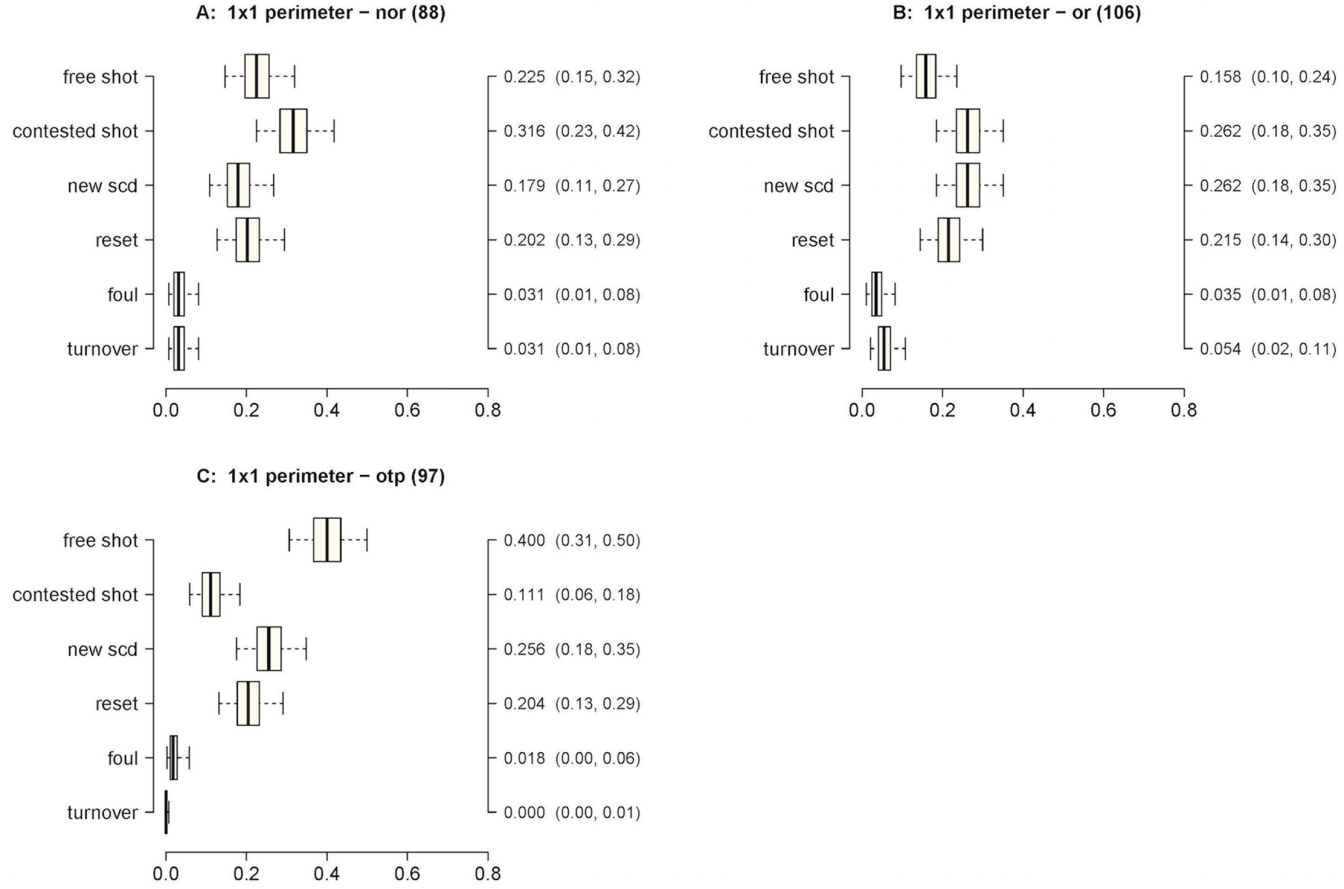
Outcome probabilities for an SCD of 1 on 1 in the perimeter. The SPDs are: Panel A) not oriented; Panel B) oriented; Panel C) defense out of position. X-axis probability; Y-axis—possible outcomes; second Y-axis—Posterior median estimate of outcome probability (95% credible intervals).

**Fig 4:**
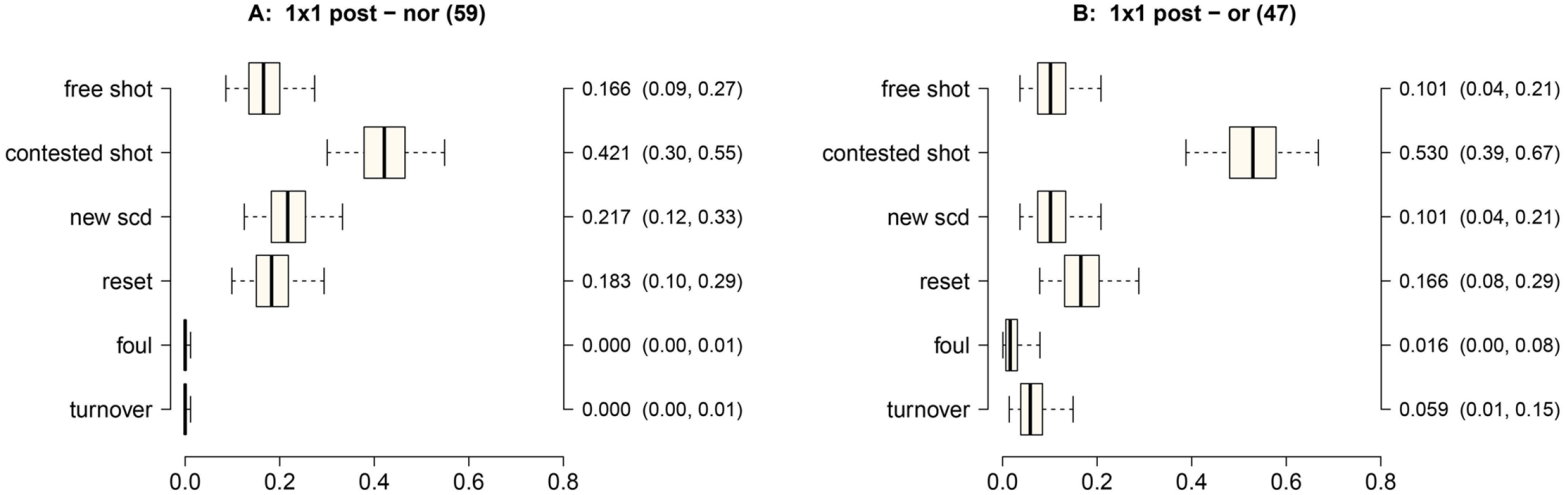
Outcome probabilities for an SCD of 1 on 1 in the post. The SPDs are: Panel A) not oriented defense; Panel B) oriented defense. X-axis probability; Y-axis—possible outcomes; second Y-axis—Posterior median estimate of outcome probability (95% credible intervals).

**Fig 5:**
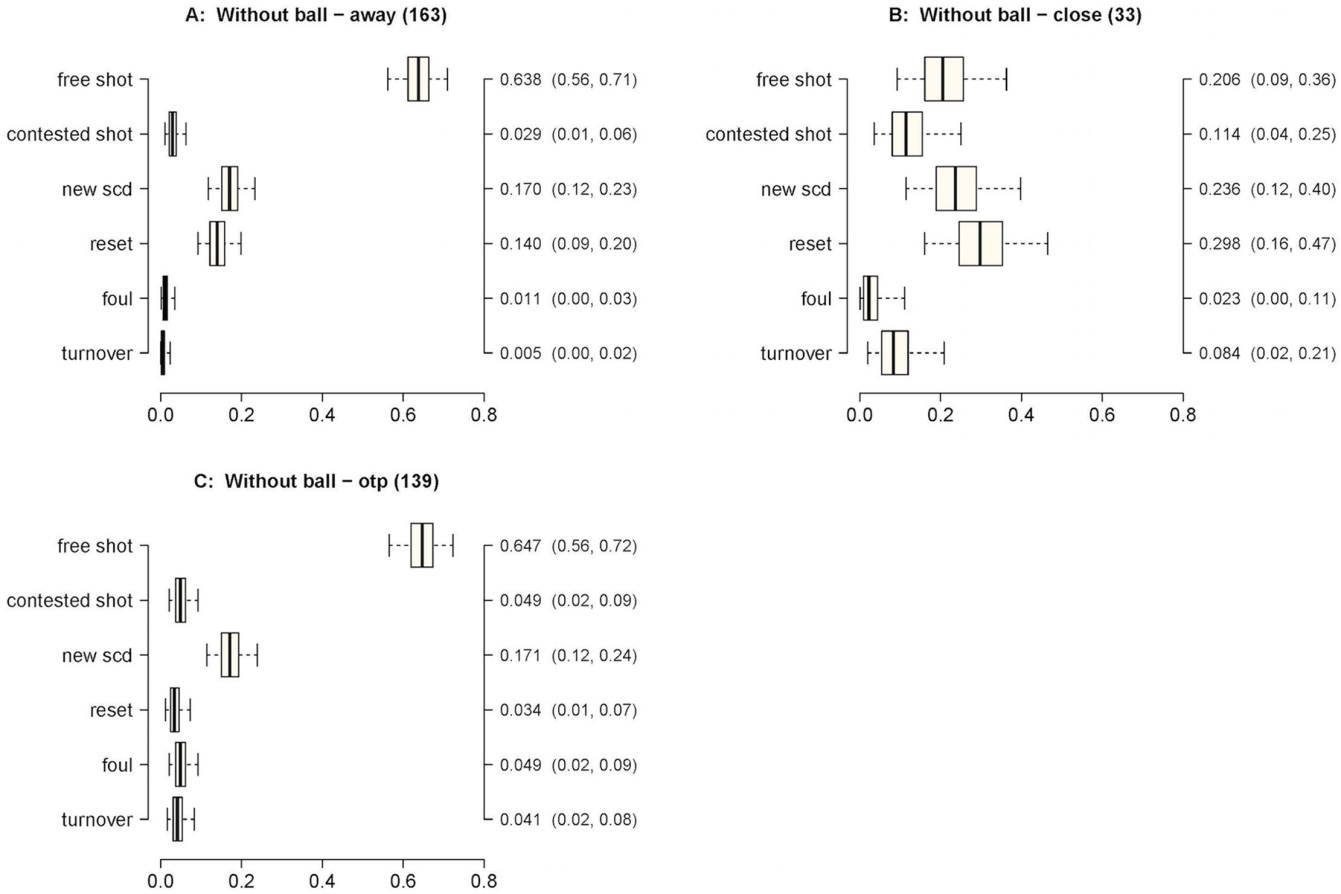
Outcome probabilities for a 2 on 2 SCD where the SPD emphasis is on the player without the ball. Panel A) away defense; Panel B) close defense; Panel C) defense out of position. X-axis probability; Y-axis—possible outcomes; second Y-axis—Posterior median estimate of outcome probability (95% credible intervals).

**Fig 6:**
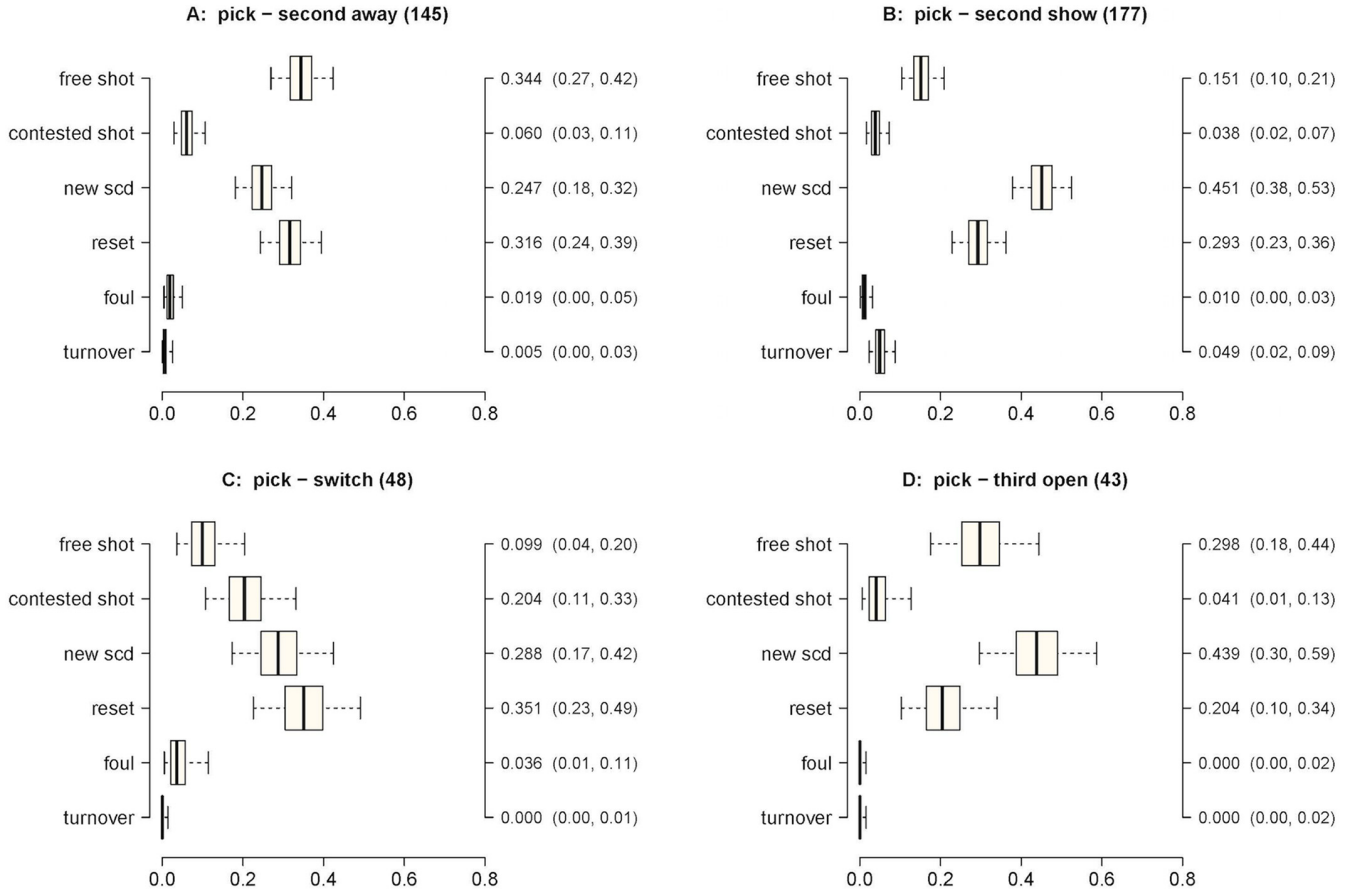
Outcome probabilities for a 2 on 2 pick. Panel A) second-away; Panel B) second-show; Panel C) switch; Panel D) third-open. X-axis probability; Y-axis—possible outcomes; second Y-axis—Posterior median estimate of outcome probability (95% credible intervals).

**Fig 7:**
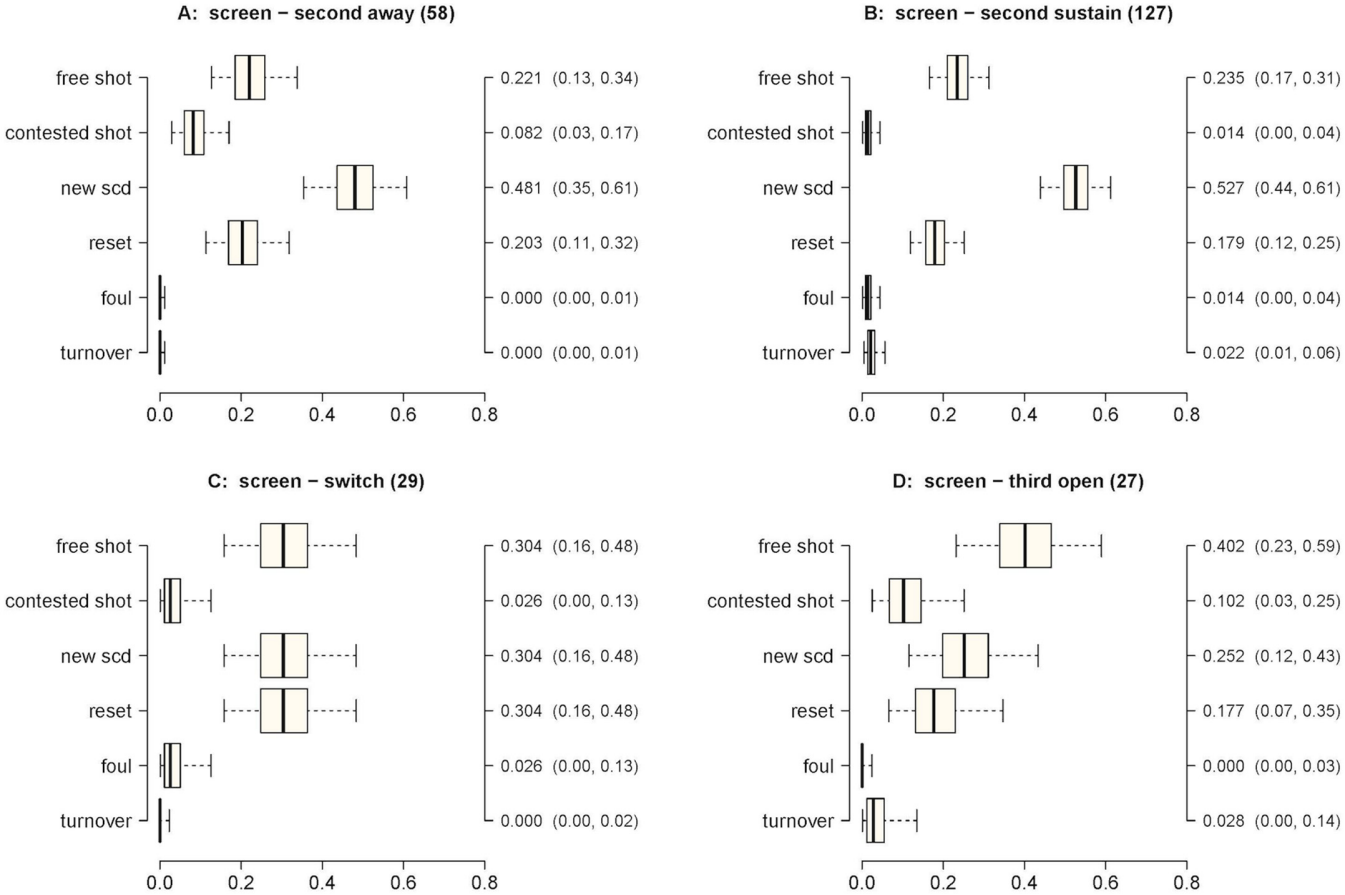
Outcome probabilities for a 3 on 3 screen. Panel A) second-away; Panel B) second-sustain; Panel C) switch; Panel D) third-open. X-axis probability; Y-axis—possible outcomes; second Y-axis—Posterior median estimate of outcome probability (95% credible intervals).

For the purpose of this paper, when a probability is classified as ‘significant’, it means the posterior probability of the stated event (*π*_post_) exceeded 0.95. We recognize that we are not using the word ‘significant’ in its classical sense, that is we are not stating and then rejecting a series of null hypotheses. Rather, since we are considering these problems from a Bayesian standpoint, we are simply attempting to categorize the strength of a posterior probability. Further, we also recognize that the generalizability of the study may be suspect, since our work focuses on one team. Nonetheless, we feel that the use of statements about the posterior probability of the various events are useful in characterizing offensive and defensive strategies in basketball. More extensive posterior summaries for several of the models are reported in Figs 3–7.

## Results

Reliabilty results for the game analyst responsible for data collection indicated a high intra-rater consistency between days (agreement level > 0.92). Initially, we present the posterior probabilities of the outcomes for each SCD. Next, the posterior probabilities of outcomes for SCD-SPD combinations are given.

The relative frequencies of the SCDs observed in the matches analyzed were the following: i) 1 on 1 perimeter—19% (diagrams 1A and 1B in Fig 1); ii) 1 on 1 post—8% (diagrams 2A and 2B in Fig 1); iii) without ball—22% (diagrams 3A and 3B in Fig 1); iv) pick—33% (diagrams 4A-6B in Fig 1); v) screen—18% (diagrams 7A-9A in Fig 1). The SCDs that produced the two highest median posterior probabilities of occurrence of the predefined outcomes were the following (see Fig 2): i) free shot—without the ball (0.409) or a pick (0.235); ii) contested shot—1 on 1 perimeter (0.367) and 1 on 1 post (0.285); iii) new SCD—a pick (0.403) and a screen (0.268); iv) reset—a pick (0.457) and a 1 on 1 perimeter (0.186); v) foul—a pick (0.329) and without ball (0.277); vi) turnover—a pick (0.366) and without ball (0.257).

Figures below present the probabilities of outcomes following SCD-SPD couples. The credible intervals are given in the box plots of each figure. Fig 3 presents the probabilities of the outcomes when the interactions start with an action of the ball player on the perimeter (i.e. 1 on 1 on the perimeter). The contested shot (0.316) was the most likely outcome when a non-oriented defense (Panel A) was used. When an oriented defense was used (Panel B), the contested shot (0.262) and new SCD (0.262) had higher probabilities of occurrence than the other outcomes. When the defense was out of position (Panel C), a free shot (0.400) had the highest probability. These data offer compelling evidence that defense out of position produced a significantly higher probability of a free shot than oriented defense (*π*_post_ > 0.99). Similarly, non-oriented defense produced a significantly higher probability of a contested shot than defense out of position (*π*_post_ > 0.99).

Fig 4 presents the probabilities of the outcomes when interactions started in the post (1 on 1 in the post). A contested shot (0.421) was the most likely outcome in the case of non-oriented defense (Panel A). Similarly, the contested shot (0.530) was the most likely outcome for oriented defense (Panel B). Both non-oriented (0.166) and oriented (0.101) defenses produced significantly lower probabilities of a free shot than a contested shot (*π*_post_ > 0.99, in both cases).

Fig 5 presents the probabilities of the outcomes when interactions started with space creation without ball. A free shot (0.638) had the highest probability of occurrence in an away defense (Panel A). The probability of a free shot was significantly lower (0.206) in a close defense (Panel B). The close defense had a significantly higher probability (0.298) of a reset than either an away defense (0.140) or an out of position defense (0.034), (*π*_post_ = 0.98 and *π*_post_ > 0.99, respectively).

In Fig 6 we present the probabilities of the outcomes when interactions started with a pick. Free shot (0.344), reset (0.316) and new SCD (0.247) had the highest probabilities among the possible outcomes with a second-away defense (Panel A). In a second-show defense (Panel B), new SCD (0.451) had the highest probability of occurrence, followed by reset (0.293). Regarding the switch defense, reset (0.351), new SCD (0.288), and contested shot (0.204) had the highest probabilities of occurrence. A new SCD (0.439) had a higher probability of occurrence than any other outcome except free shot (0.298) in the third-open SPD. Second-away (0.344) had a significantly higher probability of leading to a free shot than either second-show (0.151) or switch (0.099) (*π*_post_ > 0.99, in both cases). Alternatively, second-show (0.451) produced a higher probability of occurrence of a new SCD than either second-away (0.247) or switch (0.288) (*π*_post_ = 0.97 and *π*_post_ > 0.99, respectively). Overall, these SPDs presented low probabilities of leading to a contested shot. Finally, there were observed moderate and similar probabilities of leading to a reset among the defenses.

In Fig 7 we present the probabilities of the outcomes when interactions started with a screen. Switches produced moderate and similar probabilities (0.304 in each case) of a free shot, a new SCD, and a reset. The most likely outcome following a third-open defense was a free shot (0.402). Third-open produced a significantly higher probability of a free shot than second-away (0.221) or second-sustain (0.235) (*π*_post_ = 0.95 and *π*_post_ = 0.96). A contested shot had low and similar probabilities among the different types of defense. New SCD had a significantly higher probability for second-away (0.481) and second-sustain (0.527) than for third-open (0.252) (*π*_post_ = 0.97 and *π*_post_ > 0.99). For second-away and second-sustain the probability of a new SCD was significantly higher than any other outcome (*π*_post_ > 0.99).

## Discussion

This study aimed to model the relationship between SCDs, SPDs and outcomes obtained from the analyses of professional basketball matches. Overall, the novel finding of the present study encompasses the ability to determine SCDs favoring either positive attack outcomes (e.g. free shot) or negative attack outcomes (e.g. turnover) when taking into consideration all possible SPDs.

The presented work is complementary to several studies that have developed variables to assess the collective dynamic features of the opposition between teams [20–22]. Differently from previous studies, the semantic of the actions was the focus of the analyses and match situations were distinguished based on the variables that describe each game state in a sequence of events. This type of analysis provides criteria for selecting information that are relevant to explain teams’ tactics and its relation to team strategy.

The analysis of SCD-SPD couples showed the outcome obtained with every SCD was influenced by the type of SPD performed by the adversary. This reinforces the notion that the opposition should be analyzed taking into consideration the offensive-defensive interaction. Thus, the present study was an improvement on previous work in which only the SCDs of a single team were considered in the analysis [13] or in which a predictive modeling was missing [14].

The pick was the most prevalent SCD (33%). However, this SCD did not produce the highest probability of a free shot (0.235). The highest probability of a free shot followed a without ball SCD (0.409). The pick seemed to be used to not only lead to a scoring opportunity but also to initiate offenses, as it produced the highest probability leading to a new SCD (0.403). The without ball also presented the second greatest probability of leading to a foul (0.277) or a turnover (0.257). The mentioned probabilities of succesful outcomes (i.e. free shot or foul) and unsuccesful outcome (i.e. turnover) of the without ball characterize this SCD as possibly providing a high reward even though a non-neglectable risk also exists. Although frequent (19%), 1 on 1 perimeter presented the highest probability of a contested shot (0.367), which corroborates with the empirical notion that the difficulty for the ball player to free himself without any help increases in modern basketball. The second hightest probability of a contested shot was found for 1 on 1 post (0.285) and this SCD presented the lowest frequency of ocurrence (8%). Besides the fact that the post area is naturally well protected for its proximity to the basket, favoring the shot be contested, low recurrence of 1 on 1 post aligns with the defensive effort for keeping the ball away from the post. Finally, there was not a great difference in outcome when the SCD was a screen.

In the 1 on 1 perimeter SCD, an out of position defense yielded the greatest probability of a free shot (0.400). Out of position defense is often the consequence of a previous SCD or a defensive mistake. Hence, from an offensive standpoint, this result supports the notion that the concatenation of disruptive actions is useful in progressively creating sufficient space. Other contextual elements, such as the tactical expertise of the offensive player being guarded, may indicate which SPD should be performed.

In the 1 on 1 in the post SCD, both oriented (0.503) and non-oriented (0.421) defenses produced high probabilities of contested shots. Due to the proximity to the basket, the defense is always close in post actions, but often these contested shots have a high probability of scoring points. Additionally, in a non-oriented defense, the need to guard the post player by concentrating defensive players close to the basket often leads to a new SCD (0.217).

The without the ball SCD was more efficiently guarded by a close defense (free shot probability of 0.206) than both away defense (0.638) and out of position defense (0.647). An away defense may be a consequence of the displacement of a defensive player to another area of the court to help a teammate. Also, an out of position defense may represent a stronger commitment of one defender in helping another one. The outcomes of these SPDs against a without ball SCD highlight a strategic concern regarding help defense, as the commitment in helping other defensive players seems to reduce the ability of the defenders to guard their assigned attackers.

In relation to the pick SCD, the second-show SPD (0.151) was significantly more effective than either the second-away (0.344) or the third-open SPD (0.298) in decreasing the probability of free shots (*π*_post_ > 0.99 and *π*_post_ = 0.98, respectively). Among these three SPDs, second-show puts the greatest pressure on the ball player. All three of these SPDs, second-show, second-away and third-open, lead to a reset with similar probabilities (0.293, 0.316 and 0.204). Second-show also yielded a high probability of leading to a new SCD (0.451), indicating a possible trade-off between avoiding a shot and enabling the offense to concatenate a new SCD because of the high commitment of the defenders involved. The defender performing the show is momentarily allocated in the defense of the ball player and his attacker may move to start a new SCD.

In regards to the screen, the third-open SPD led to the highest probability of free shots (0.402). This is expected because this SPD provides enough space for the screen receiver to perform an immediate shot. For the other SPDs, second-away and second-sustain, there was a high probability of a new SCD occurring, 0.481 and 0.527 respectively. The high probability of a new SCD seems to be due to the defensive demands in guarding an offensive action involving three players, which requires the commitment of the defenders in helping each other.

In the present study, the sample data were gathered from three high-level basketball teams only. Thus, one should consider this fact when attempting to generalize the practical findings in the present study to other teams or leagues. Nonetheless, previous work has shown that similar situations arise at different competition levels indicating that our findings may be applicable to different samples [23]. Additionally, considering the current technological improvements that have enabled acquiring larger amounts of data from a game or even a complete season [24], these findings may be helpful for labelling events on a large scale supported by pattern recognition methods.

In summary, to the best of our knowledge, in team sports, this was the first study to model the tactical features of the offense-defense interaction. Our analyses revealed that SCDs with high frequency of occurrence did not necessarily lead to high probabilities of free shots. However, SCD’s often lead to a new SCD as an outcome, evidencing offensive strategic features of progressive disruption of the defensive system through the concatenation of subsequent offensive actions.

## Supporting Information

S1 Dataset Triples of SCD-SPD-outcome from the sample. (XLSX)
